# Development of copper based drugs, radiopharmaceuticals and medical materials

**DOI:** 10.1007/s10534-012-9578-y

**Published:** 2012-08-23

**Authors:** Paweł Szymański, Tomasz Frączek, Magdalena Markowicz, Elżbieta Mikiciuk-Olasik

**Affiliations:** Department of Pharmaceutical Chemistry and Drug Analysis, Medical University of Lodz, Muszyńskiego 1, 90-151 Lodz, Poland

**Keywords:** Copper, Nuclear medicine, Nanotechnology, Drug development

## Abstract

Copper is one of the most interesting elements for various biomedical applications. Copper compounds show vast array of biological actions, including anti-inflammatory, anti-proliferative, biocidal and other. It also offers a selection of radioisotopes, suitable for nuclear imaging and radiotherapy. Quick progress in nanotechnology opened new possibilities for design of copper based drugs and medical materials. To date, copper has not found many uses in medicine, but number of ongoing research, as well as preclinical and clinical studies, will most likely lead to many novel applications of copper in the near future.

## Introduction

Copper (Cu) is a transition metal with atomic number 29, known since ancient times. It is an important trace element for most organisms in all kingdoms. In humans, copper plays role as a cofactor for numerous enzymes, such as Cu/Zn-superoxide dismutase, cytochrome *c* oxidase, tyrosinase, ceruloplasmin and other proteins, crucial for respiration, iron transport and metabolism, cell growth, hemostasis (Puig and Thiele 2002; Bertini et al. 2010). With the progress in medical sciences, copper has gained a lot of attention. The number of publications concerning copper and its compounds for potential medical applications, have reached tens of thousands. There are several reasons that render this element so attractive for drug development. Generally, simple inorganic salts of copper are toxic, but as a transition metal, with unsaturated *d* shell, it forms a large number of complexes. Coordination chemistry of copper is well-studied and “straightforward” in comparison to many other elements. From three known oxidation states, +1 and +3 are mostly unstable in biological systems, but on +2 state, Cu forms stable complexes with coordination number of 4, 5 or 6. Administration of copper in a form of organometallic complexes can be done in order to selectively deliver copper ions or radionuclides to diseased tissues, or to modify pharmacokinetics and/or pharmacodynamics of ligands. Moderate amounts of metal ions that could be liberated from biological degradation or transchelation of Cu complexes can be managed by organism, as copper is an important microelement, in contrary to many other transition metals, whose leakage from their compounds can lead to accumulation and toxic effects. Copper has several radioisotopes, five of them are particularly interesting for radiotherapy and imaging applications. Continuous progress of nanotechnology made it possible to exploit novel physicochemical properties of copper-containing nanoparticles and molecules. This article reviews current trends in various fields of medicine, in development of copper based pharmaceuticals and medical materials.

## Biological activity of complexes of stable copper isotopes

### Inflammation

In folklore it is believed that wearing copper bracelets and jewellery can ease the pain in rheumatoid arthritis. This belief had drawn attention to possible anti-inflammatory properties of copper ions and complexes. This issue was extensively researched in past century by Sorenson (1976, 1982, 1987, 1989). Hostýnek et al. (2006) found that metallic copper can indeed penetrate skin, after being oxidized on air. Anti-inflammatory effect of Cu can be linked with modulation of prostaglandin synthesis (Sakuma et al. 1996; Franco and Doria 1997; Sakuma et al. 1999), interleukin IL-2 expression (Hopkins and Failla 1999), neutralization of reactive oxygen radicals by Cu/Zn-superoxide dismutase and other. Though copper deficiency is known to impair immunity, the exact mechanism is unclear (Huang and Failla 2000).

In the past decade, several authors reported copper(II) complexes with potential anti-inflammatory properties. For treatment of rheumatoid arthritis, chelating agents that can facilitate transport of Cu(II) ions to sites of inflammation were researched (**1**–**13**).
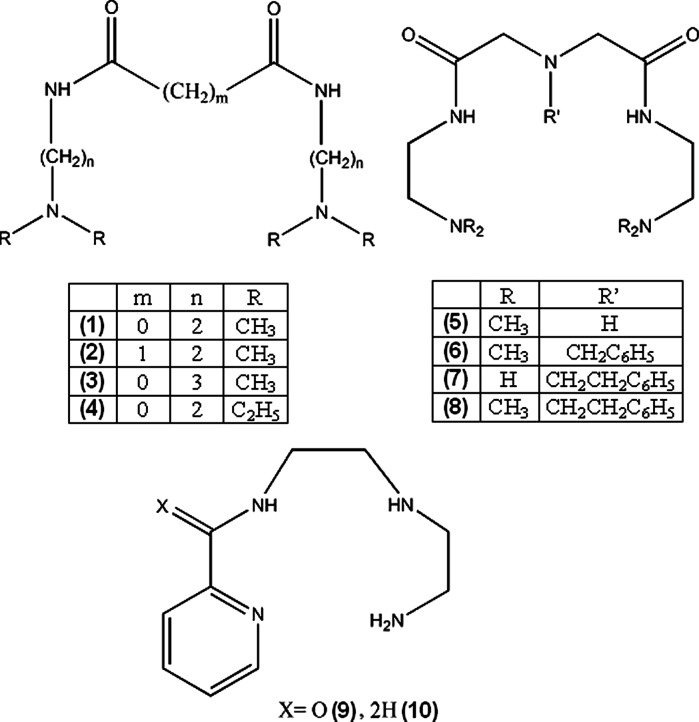


Jackson et al. (2000) attempted to design linear polyamine ligands that can mobilize copper in organism. The complexes cannot be too stable, because they would be quickly excreted with urine in unchanged form. Ligands **1**–**4** formed neutral complexes only above pH 7.0 and were too labile for systemic administration, but still could be used to facilitate dermal absorption of copper. Complexes of **5**–**8**, due to additional nitrogen atom were significantly more stable (~2 log units), **6**–**8** were also more lipophilic, but the stability was still suboptimal (Jackson et al. 2000). More promising results for dermally absorbed Cu complexes were achieved for ligands **9** and **10**. The compounds show selectivity towards copper ions, good stability at physiological pH (formation constants at 25 °C in 0.15 M NaCl, for unprotonated ligands: log β = 11.51 for **9** and 18.62 for **10**), low renal clearance and water/octanol partitioning indicating possible dermal absorption. An important feature of **9** and **10** is that they form more labile complexes with Ca^2+^ and Zn^2+^ ions (for **9** and **10** respectively: with zinc log β = 5.55 and 11.51, with calcium log β = 3.24 and 3.92), which are main competitors of copper in blood plasma. Simulations showed that Cu complexes of the ligands are stable in blood plasma, and effectively mobilize copper ions without affecting significantly other metal ions levels (Zvimba and Jackson 2007). Odisitse et al. (2007, 2009) also reported dermally absorbed complexes of copper with **11**–**13** ligands. The compounds showed approximately 24 h biological half-life which is desired for potential anti-inflammatory drugs. Simulation of behavior of **13** in blood plasma indicated that Ca^2+^ and Zn^2+^ ions concentration is sufficient to compete with Cu^2+^, even though **13** is more selective towards cupric ions. Therefore, the ligand can facilitate copper transport through skin, then release Cu^2+^ ions in bloodstream.
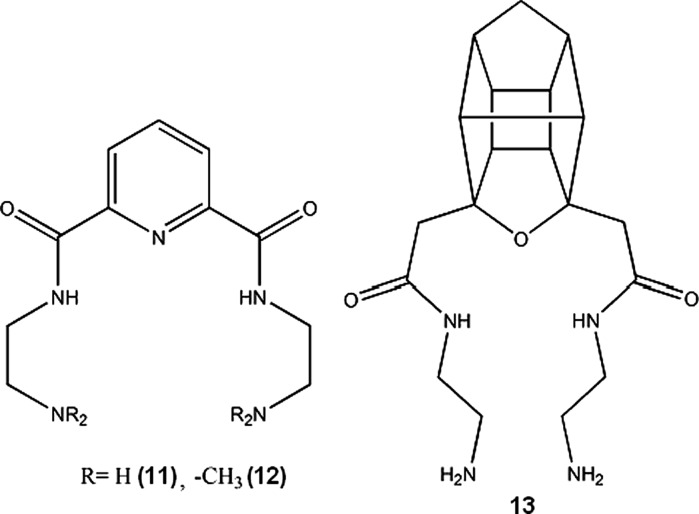


Copper-zinc-superoxide dismutase (SOD) is an important enzyme protecting cells against oxidative injury, scavenging and neutralizing reactive oxygen species. It has been shown that SOD can significantly reduce inflammation induced in laboratory animals (Emerit et al. 1991; Zhang et al. 2002; Garcia-González et al. 2009). Many complexes of copper(II) have similar to SOD ability to neutralize superoxides (**14**–**27**). These SOD-mimicking complexes of copper were proposed as non-analgesic anti-inflammatory drugs by various authors: Cu complexes of aromatic acids (**14**–**16**) (Suksrichavalit et al. 2008), saccharinate and pyridine derivates (**17**–**18**) (Ferrer et al. 2010), tolfenamic acid (**19**) (Kovala-Demertzi et al. 2004), 2-amino-2-thiazoline and polyamines (**20**–**25**) (Pontiki et al. 2006), o-vanillin (**26**) (González-Baró et al. 2010), oxaprozinate (**27**) (Dutta et al. 2004).
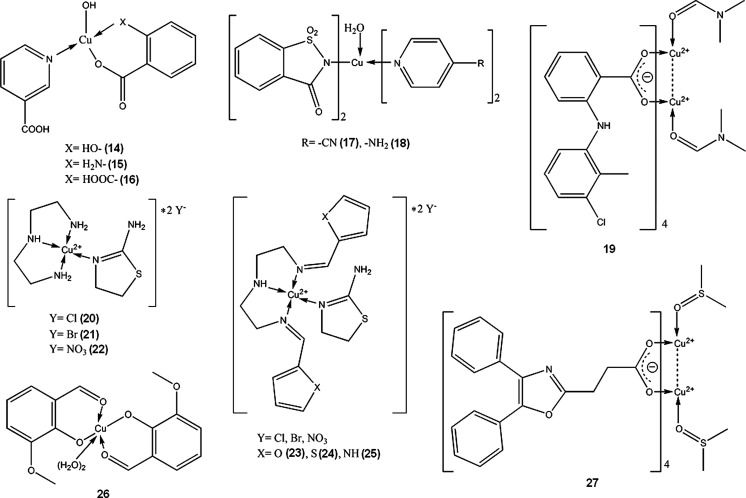


However, there is one important hindrance, as both SOD and SOD-like Cu-complexes can cleave cellular DNA by reacting with hydrogen peroxide and generating hydroxyl radicals by Fenton-type reaction (Sang and Yang 2005; Han et al. 2007; Seng et al. 2009; Ghosh et al. 2010; Ibrahim et al. 2011). This aspect requires thorough dose–response studies, before this group of potential anti-inflammatory drugs can emerge.

In the last 20–30 years of 20th century, there was a lot of interest in copper(II) complexes with NSAIDs (Non-Steroidal Anti-Inflammatory Drugs), such as acetylsalicylic acid, indomethacin, piroxicam, ibuprofen, diclofenac, naproxen and others. These complexes were reported to possess increased activity and lower ulcerogenic effect than respective NSAID and copper administered separately. However, none of the substances has been approved for internal therapy of humans. Exhaustive review of the subject was written by Weder et al. (2002) Currently, there are still a number of publications each year on Cu-NSAIDs complexes, but mainly concerning the structural and physicochemical aspects of the compounds; structure–activity and biological studies are sparse, therefore it can be assumed that this group of potential drugs will not make any impact on medicine in the nearest future.
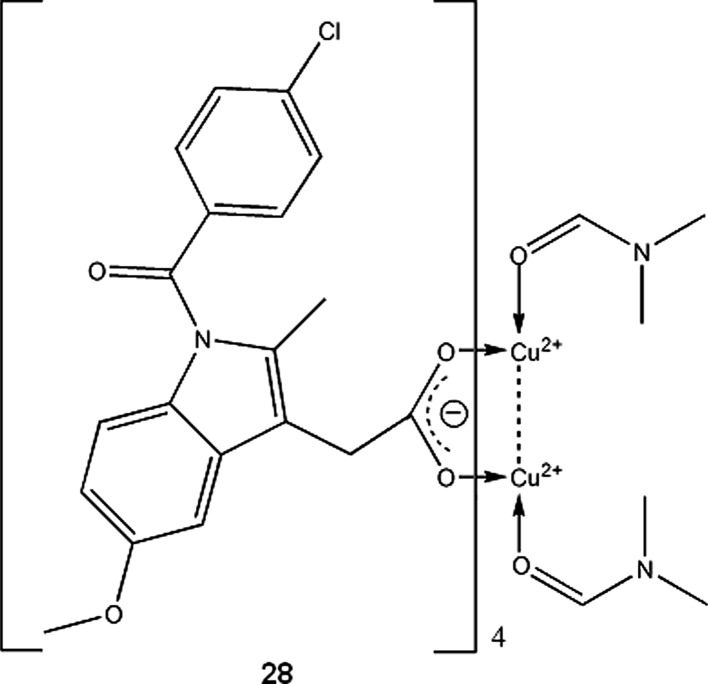


**Fig. 1:**
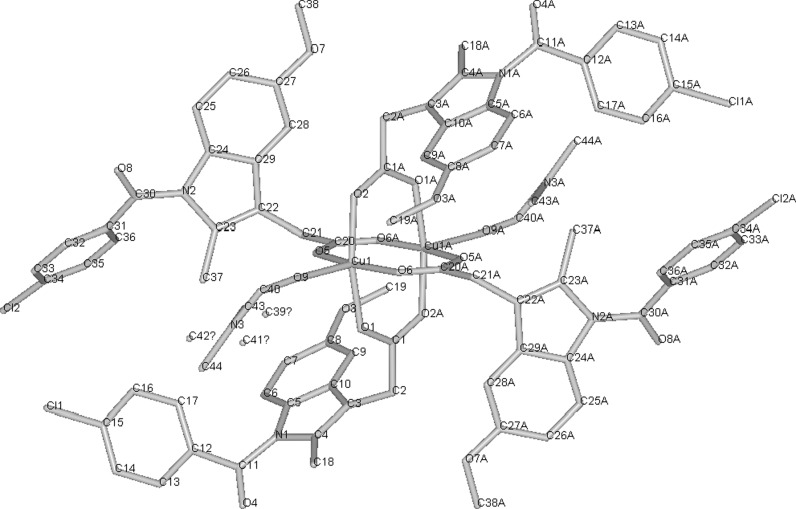
Copper-indomethacine-N,N-dimethylformamide complex (Weder et al. 1999). Data from Cambridge Crystallographic Data Centre

It should be noted that copper-indomethacine complex (**28**, Fig. 1) underwent some biological evaluation, and is currently used in veterinary in Australia, New Zealand and some other countries. Similarly to other NSAID-Cu complexes, copper indomethacinate retains parent drug anti-inflammatory activity but have lower ulcerogenic effect, probably due to free radical scavenging ability (Bertrand et al. 1999). Cu-indomethacin-dimethylformamide complex shows good solution stability at pH 7.4 (<8 % decomposition after 3 days), which can be further increased by micellar solubilisation of the complex with Span 80 and tetraglycol (Weder et al. 1999). In Australia, Cu-salicylate was available until recently for external use in humans, in a form of topical anti-inflammatory gel.

### Cancer

**Fig. 2:**
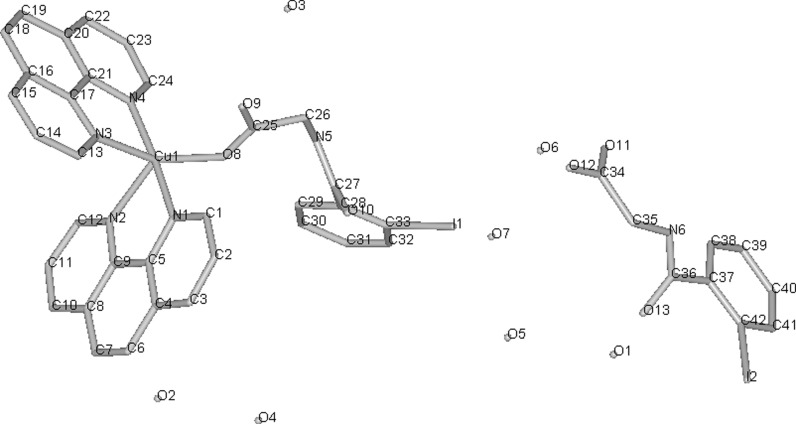
Crystal structure of Cu-*o*-iodohippurate-1,10-phenantroline complex(**29**) (Barceló-Oliver et al. 2007) Data from Cambridge Crystallographic Data Centre

Since cisplatin was introduced for chemotherapy of cancer, a search for other transition metal complexes with anti-proliferative activity has started. Various copper(II) complexes were found to be cytotoxic, with the most common ligands being NSAIDs or Schiff bases (**29**–**38**). As mentioned in above section, many Cu(II) complexes possess catalytic activity towards reactive oxygen species and can induce breakage of DNA strand. This can explain cytotoxicity of some of the compounds. **29** (Fig. 2) in aqueous solution without presence of any external reducing factors, forms bis-(1,10-phenantroline)copper(I) which oxidatively degrades nucleic acids (Barceló-Oliver et al. 2007). However, in many cases, probably more sophisticated mechanisms are involved.

**Fig. 3:**
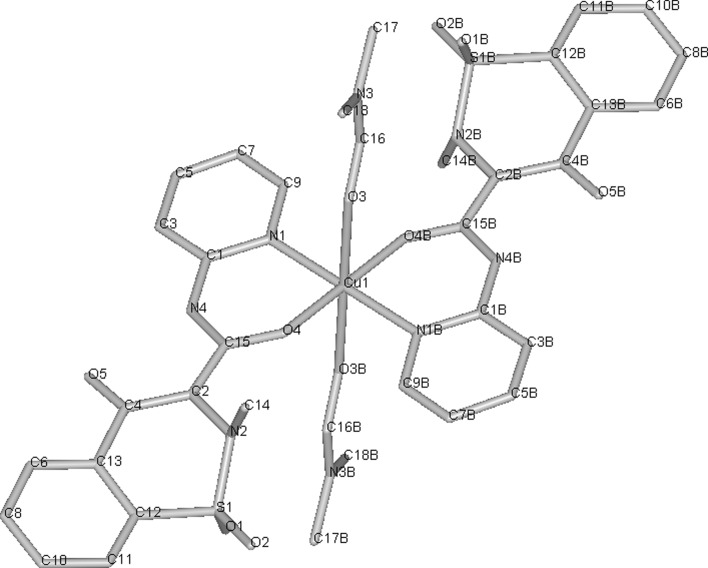
Copper piroxicam-DMF complex crystal structure (Cini et al. 1990) Data from Cambridge Crystallographic Data Centre

Meloxicam (**30**) and piroxicam (**31**, Fig. 3) form stable in physiological pH Cu complexes (K = 3.2 × 10^9^ and 9.8 × 10^9^ M^−2^ respectively) that are able to strongly bind with DNA, disrupting its structure and stopping transcription as a result (Roy et al. 2006; Cini et al. 2007). Copper N-(2-hydroxyacetophenone) glycinate (**32**) is an interesting immunomodulatory agent, capable of inducing apoptosis in multidrug-resistant cancer cells by stimulating production of cytokines, such as interferon γ or TNF-α (Tumor Necrosis Factor α) (Mookerjee et al. 2006). Guo et al. (2010) suggested that salicylaldehyde-amino acid Schiff base copper chelates (**33**, **34**) trigger cancer cell’s apoptosis by downregulation of overexpressed mutant type P53 protein.
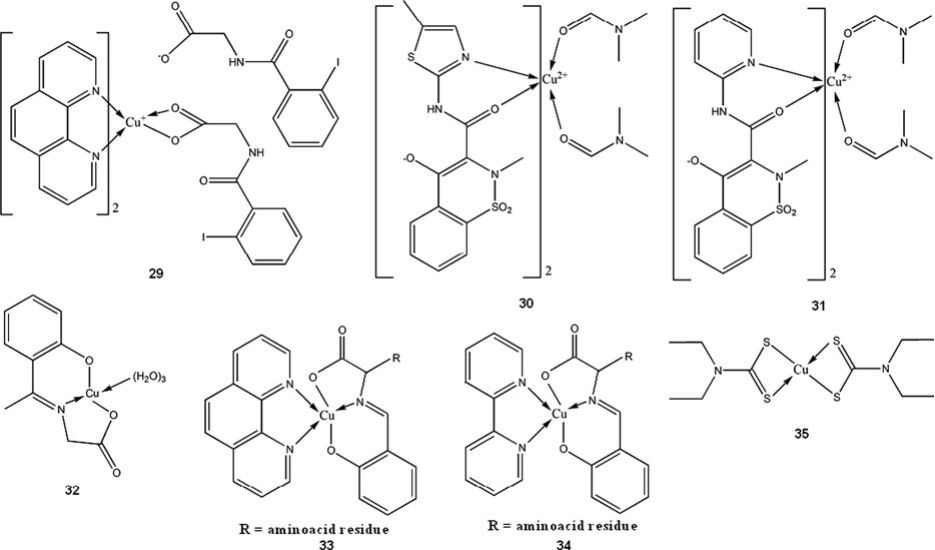


Disulfiram, a drug used in alcoholism treatment, forms in vivo a copper complex (**35**) which acts as a proteasome inhibitor, and selectively induces apoptosis in breast tumors (Chen et al. 2006). Disulfiram and copper gluconate are currently under phase I trials for treatment of solid tumors with metastases in liver (ClinicalTrials.gov 2012). Compound **36** shows high in vitro and in vivo activity towards MCF-7, PC3 and HEK293 cell lines. Its proposed mode of action is multidirectional and includes apoptosis induction via caspase pathway, DNA fragmentation and antioxidant enzymes inhibition. **36** is more effective than cisplatin in breast tumor models (about 20-fold lower IC_50_) and shows minimal toxicity (Chakraborty et al. 2010). Other type Cu(II) complexes with both antimicrobial and antitumor properties were reported by Singh et al. (2009)(**37**,**38**).
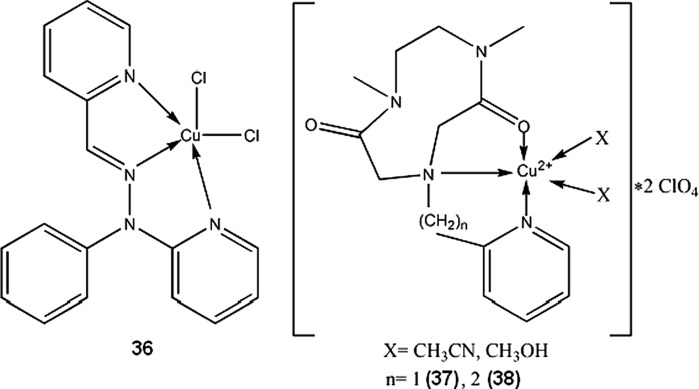

### Antimicrobial

Copper, both in metallic form and in many chemical compounds, possess antimicrobial activity, which was already used by ancients. Cupric ions exhibit non-specific biocidal activity, although weaker than silver. Copper-silver electrolytic ionization systems are used in many hospitals to decrease number of *Legionella* residing in hot water pipes. Metals and alloys used in orthopedic implants can be doped with copper ions, in order to reduce risk of infection after prosthetic surgery. The tradeoff is reduced to some extent corrosion resistance of the resulting materials, but still on a reasonable level (Wan et al. 2007). Due to non-specific toxicity, for the use of copper as an antibacterial therapeutic, the metal should be administered in a form of complex compounds, rather than simple inorganic salts. Nature of chelating agent, however, plays very important role, as there can be no simple correlation between antibacterial activity and complex stability (Azenha et al. 1995). Many various Cu(II) complexes with different ligands were reported to possess antibacterial and antifungal activity (**39**–**48**) (Gölcü et al. 2005; Shakir et al. 2006; Singh et al. 2008; Sreedaran et al. 2008; Kumar and Arunachalam 2009; Suksrichavalit et al. 2009). Singh et al. (2008) utilized an approach to use ligands which already have antimicrobial activity and enhance it by complexation with copper (**39**–**41**). Antihypertensive drug pindolol, when complexed with Cu (**41**) (complex stability constant log β = 11.28 in water-dioxan 40:60 at 25 °C), exhibits notable antimicrobial activity towards some bacterial and fungal strains (Gölcü et al. 2005). Water soluble, polymeric complex **47** shows good antimicrobial activity and is also capable of binding DNA (Kumar and Arunachalam 2009). The complexes (**39**–**48**) were only screened for antibiotic properties, and to the best of our knowledge no further evaluations for medical applicability were performed.
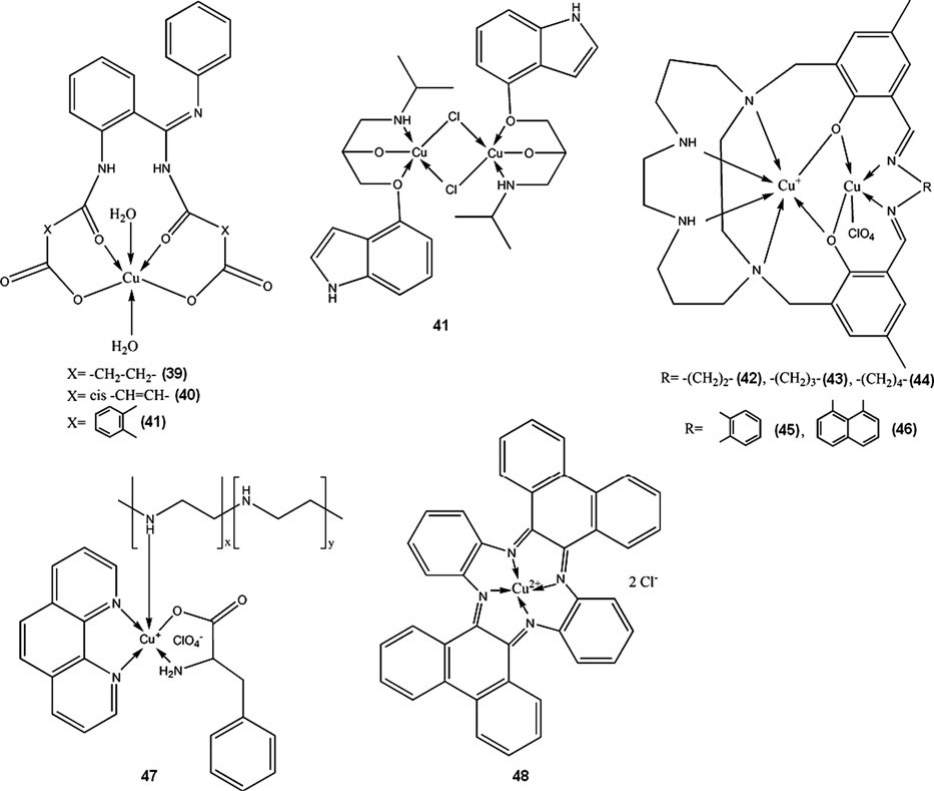


### Other uses

Copper (I)-Cl-(nicotinic acid)_2_ (polymeric) is able to notably reduce gastrointestinal mucosa lesion caused by NSAIDs such as acetylsalicylic acid. The complex shows antioxidative, antiapoptotic, secretolytic and antihemorrhagic activity and can be a good alternative for currently used anti-ulcer drugs, proton pump inhibitors, which increase gastrin level (Tuorkey and Abdul-Aziz 2009). It is also a rare example of Cu(I) compound proposed for medical use. Toyota et al. (2005) described a series of copper and iron complexes acting as thrombin inhibitors. One of these compounds, Cu(II) complex with 4-formyl-3-hydroxybenzamidine and d-tryptophane (**49**), had the highest inhibitory activity (K_i_ value 2.7 × 10^−8^ M), comparable to registered anticoagulant drug, argatroban (K_i_ 1.9 × 10^−8^ M) (Toyota et al. 2005). Tian et al. (2009) suggested copper-taurine as possible compound able to facilitate wounds healing by stimulating process of tissue regeneration and by preventing infections.
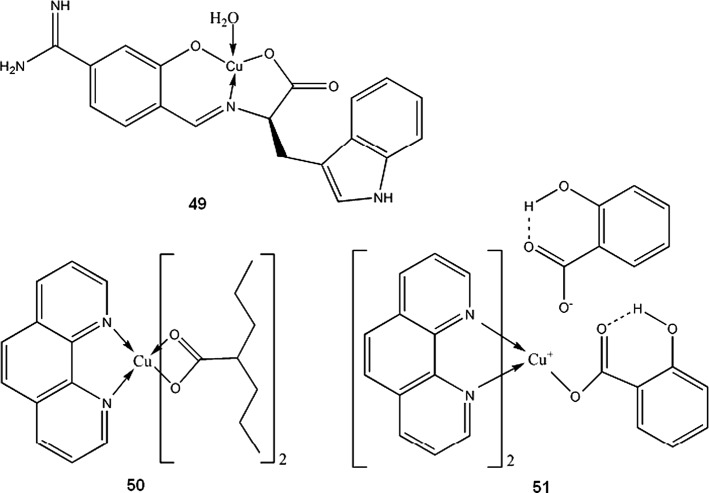


**Fig. 4:**
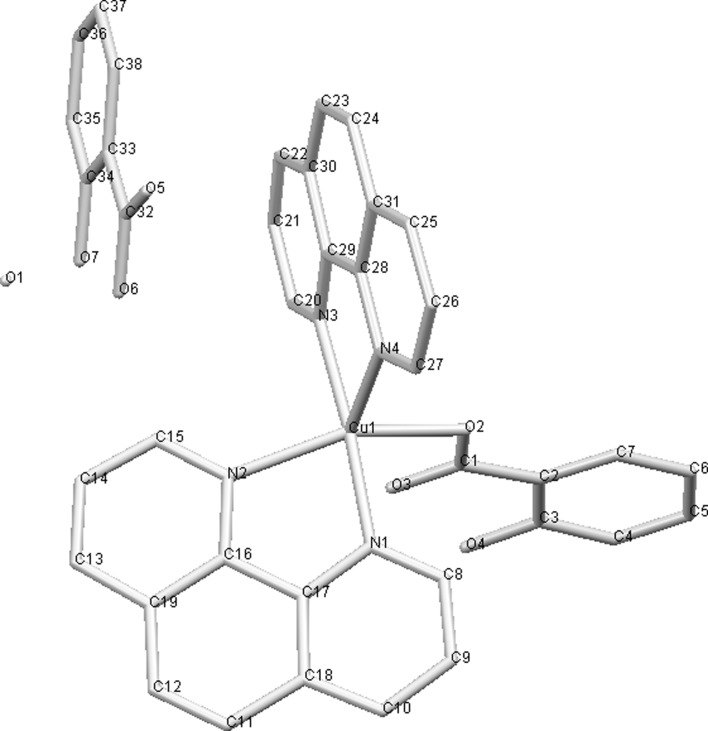
Crystal structure of **51** (Lemoine et al. 2002) Data from Cambridge Crystallographic Data Centre

Work by Sylla-Iyarreta Veitía et al. (2009) is a good example, how complexation with copper of clinically used drug, valproic acid in that case, can lead to novel, more potent compound. Bis-valproinato(1,10-phenanthroline)copper(II) (**50**) was found to be very effective in preventing Minimal Clonic seizures (ED_50_ 8 μmol/kg). 1,10-phenantroline and salicylate Cu complex (**51**, Fig. 4) and bis(1,10-phenanthroline)-μ-bis(salicylato)dicopper(II) with anticonvulsant activity effective against MES (maximal electroshock) induced seizures, were reported earlier by Lemoine et al. (2002). Despite different structure in solid state, both complexes showed similar anticonvulsant activity, probably due to formation of the same species in the dilute solutions. The compounds lose salicylate and one phenantroline ligand in dilute *N,N*-dimethylformamide (DMF) solution to form [Cu(1,10-phenantroline)DMF_4_]^2+^. The results can only be interpolated to biological systems, since both complexes are insoluble in water.

Copper palmitate may be useful in preventing skin photosensitivity induced by porphyrins in patients who underwent photodynamic therapy. Liposomal topical cream with Cu-palmitate effectively prevented skin inflammation in photosensitized rats exposed to light (Bilgin et al. 2005). Taggar et al. (2006) successfully prepared liposomal form of anticancer drugs, topotecan and irinotecan. Copper(II) sulphate loaded liposomes accumulated and retained drug molecules due to formation of copper complex inside the liposome. The authors also reported improved therapeutic activity of this drug formulation (Taggar et al. 2006). Sreedhara et al. (2000) reported Cu-aminoglycosides complexes (of neamine and kanamycin A) as efficient deoxyribonucleases with reaction kinetics similar to enzymes. Notably, the DNA cleavage was achieved by hydrolytic pathway, without generation of free radicals (Sreedhara et al. 2000). Copper-l-histidine complex (**52**) is in phase II clinical trials for treatment of Menkes disease, a genetic disorder in Cu transport, leading to copper deficiency (ClinicalTrials.gov 2012).
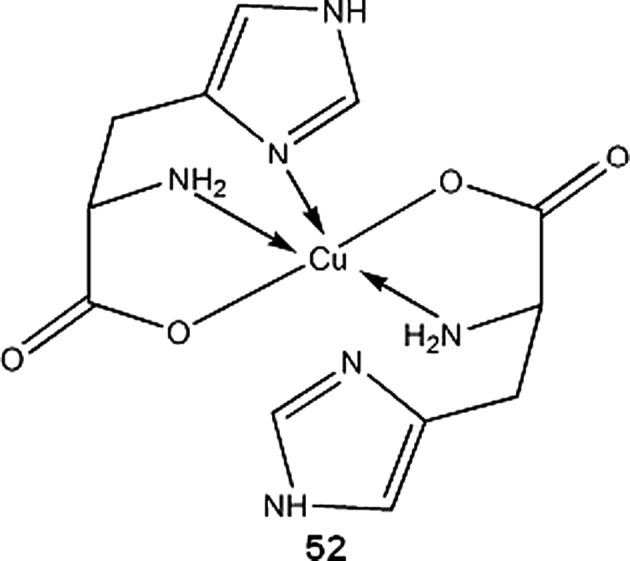


## Copper radioisotopes in nuclear medicine

Natural copper comprises two stable isotopes: ^63^Cu (69.17 %) and ^65^Cu (30.83 %). Of 27 known copper radioisotopes, five are particularly interesting for nuclear medicine: ^60^Cu, ^61^Cu, ^62^Cu, ^67^Cu, and especially ^64^Cu. Their nuclear characteristics are given in Table 1.

**Table 1:** Decay properties of medically important Cu radioisotopes

Isotope	T_1/2_	β^−^ (MeV)	β^+^ (MeV)	EC (%)	γ (MeV)
^60^Cu	23.7 min	–	1.91 (11.6 %) 1.98 (49 %) 2.95 (15 %) 3.77 (5 %)	7.2	0.511 (185 %) 0.826 (21.7 %) 1.33 (88 %) 1.79 (45.4 %) 3.12 (4.8 %)
^61^Cu	3.33 h	–	0.932 (5.5 %) 1.22 (51 %)	36	0.283 (12.2 %) 0.373 (2.1 %) 0.511 (123 %) 0.656 (10.8 %) 1.19 (3.7 %)
^62^Cu	9.67 min	–	2.93 (97.2 %)	2	0.511 (195 %)
^64^Cu	12.7 h	0.579 (38.5 %)	0.653 (17.6 %)	40	0.511 (35.2 %) 1.35 (0.5 %)
^67^Cu	61.83 h	0.377 (57 %) 0.468 (22 %) 0.562 (20 %)	–	–	0.093 (16.1 %) 0.185 (48.7 %) 0.3 (0.8 %)

Values taken from National Nuclear Data Center (Brookhaven National Laboratory 2012)

β^−^, β^+^, γ—electron, positron and gamma emission respectively, EC-electron capture

Decay characteristics of copper radionuclides make them suitable for numerous medical applications, such as Positron Emission Tomography(PET) imaging, radioimmunological tracing and radiotherapy of cancer. For widespread use in medicine of any radioisotope, two factors are essential: availability of the isotope and effective modes of binding with an appropriate chemical carrier. Efficient production of copper isotopes was extensively researched over past 20–30 years, and also many potential chelators were developed during that time. Methods of production, applications in nuclear medicine and chelating agents for copper radioisotopes were reviewed by Blower et al. (1996), Williams et al. (2005), Rowshanfarzad et al. (2006), Hao et al. (2009), Wadas et al. (2010), and Ding et al. (2011).

### ^60^Cu

PET is a three dimensional imaging technique which utilizes simultaneous detection of two oppositely moving photons, resulting from annihilation of positron with electron. Positron comes from decay of a radioisotope incorporated into targeting molecule which can selectively accumulate in desired tissues, organs or tumors. The most popular PET tracer is ^18^F in a form of 2-deoxy-2-(^18^F)fluoro-d-glucose. Metallic radioisotopes have advantage over fluorine-18, as they can be easily introduced into a targeting molecule by forming a coordination compound with it. ^60^Cu is a β^+^ emitter with decay properties making it possible candidate for PET tracer. ^60^Cu can be produced using small cyclotrons at relatively low costs from ^60^Ni target (McCarthy et al. 1999). Relatively high energy positron and gamma emissions, compared to ^62^Cu, are the most important disadvantages of ^60^Cu isotope as PET imaging agent.

Copper bis-thiosemicarbazones complexes, mainly ^60/61/62/64^Cu-diacetyl-bis(N^4^-methylthiosemicarbazone) (^60/61/62/64^Cu-ATSM **53**), ^60/61/62/64^Cu-pyruvaldehyde-bis(N^4^-methylthiosemicarbazone) (^60/61/62/64^Cu-PTSM **54**) and ^60/61/62/64^Cu-ethylglyoxal bis(thiosemicarbazone) (^60/61/62/64^Cu-ETS **55**), are the most widely studied copper radioisotopes compounds for use in PET. ^60/61/62/64^Cu-ATSM and ^60/61/62/64^Cu-ETS, due to their specific redox properties, can be useful for detection and imaging of hypoxic tumor cells. Mechanism of action of copper-thiosemicarbazones in broad outline is as follows: the complex enters cell where it is spontaneously reduced from Cu(II) to Cu(I) state, then it can either be reoxidized by molecular oxygen and diffuse from the cell, or in hypoxic conditions, it irreversibly decomposes and stays trapped within cell (Dearling and Packard 2010). It should be noted that nonradioactive Cu-ATSM has been recently found to be neuroprotective agent, and can be used for Parkinson’s disease treatment (Hung et al. 2012).
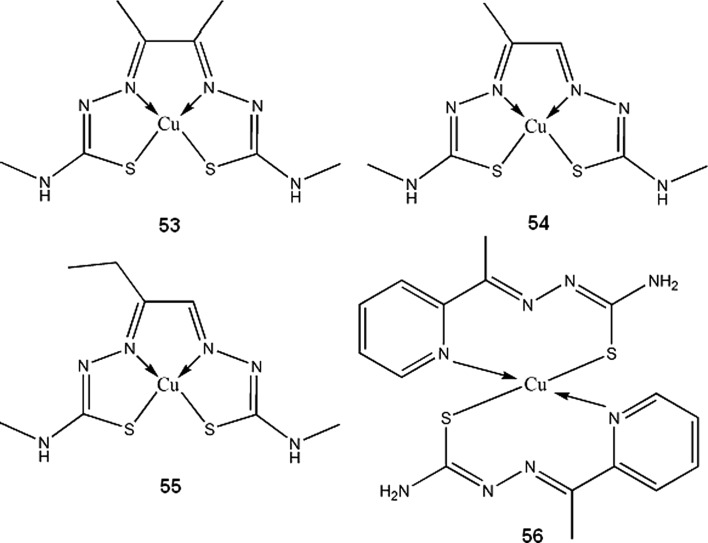


^60^Cu-ATSM was clinically studied for monitoring tumor hypoxia in lung and cervical cancer, and found to be feasible for prediction of tumor response to therapy (Dehdashti et al. 2003; 2008). Analogous pilot clinical study for rectal cancer was carried by Dietz et al. (2008), and also confirmed possible applicability of ^60^Cu-ATSM. Chao et al. (2001) suggested that PET images obtained with ^60^Cu-ATSM can be used for intensity-modulated radiation therapy of head and neck cancer. Since hypoxia of the tumor makes it resistant to radiotherapy, localization with ^60^Cu-ATSM can be used to accurately deliver higher radiation doses needed for destroying cancer cells (Chao et al. 2001).

### ^61^Cu

^61^Cu isotope can be produced from zinc, nickel or cobalt targets. Necessity of highly enriched Ni and Zn targets or high energy particle beams limited accessibility of ^61^Cu for biomedical use, until more economic production methods from natural Zn or Co were developed (Rowshanfarzad et al. 2006; Hao et al. 2009; Das et al. 2012). Longer half-life than that of ^60^Cu and ^62^Cu makes ^61^Cu better choice for prolonged imaging of processes with slower kinetics. This isotope, however, is much less popular in today’s biomedical studies than the other copper radioisotopes.

^61^Cu-APTS (2-acetylpyridine thiosemicarbazone) complex (**56**), for PET imaging of cancer, was proposed by Jalilian et al. (2006) Using pyridine thiosemicarbazone as a ligand, can give additional antiproliferative activity to the compound, which was previously observed by other authors (Belicchi-Ferrari et al. 2005). Hao et al. (2009) found ^61^Cu-1,4,7,10-tetraazacyclododecane-1,4,7,10-tetraacetic acid (DOTA)–human serum albumin to be good blood pool imaging agent and suggested its application in antiangiogenic therapy monitoring. Novel approach for therapy of multinodular goitre, using human chorionic gonadotropin (hCG) directly labeled with ionic ^61^Cu (or some other β^+^ emitters) was proposed by Maiti et al. (2011) Initial studies indicates that copper-hCG complex half-life is shorter than that of a hCG –TSH (thyroid-stimulating hormone) receptor complex, thus hyperactive thyroid cells can be destroyed before internalization of the receptor occurs. More in vitro and in vivo studies are required to assess usefulness of this therapy.

### ^62^Cu

^62^Cu has unique properties being almost pure β^+^ emitter(97.2 %) with short half-life of 9,67 min. It is easily obtainable from ^62^Zn/^62^Cu generators (Fukumura et al. 2006; El-Azony 2011), however relatively short half-life of parent ^62^Zn makes these generators operable for not more than three days. This isotope is currently the most intensively studied copper radioisotope besides ^64^Cu. ^62^Cu-PTSM is extensively researched ^62^Cu radiopharmaceutical that can be used for monitoring renal, myocardial and cerebral perfusion. Mathias et al. (1995) observed high species dependent variability in binding ^62^Cu-PTSM and ^62^Cu-ATSM by serum albumin. This can render problems when predicting behavior of copper thiosemicarbazones in human system, basing on animal data. ^62^Cu-ETS (**55**) complex is proposed as an alternative to ^60/61/62/64^Cu-PTSM for PET perfusion imaging (Mathias et al. 1995; Green et al. 2007; Basken et al. 2008). ^62^Cu-PTSM can be used together with ^62^Cu-ATSM to obtain complementary data on tumor hypoxia and blood circulation in a single PET session (Black et al. 2008; Wong et al. 2008). ^62^Cu-ATSM complex is widely researched for PET imaging of tumor hypoxia (Laforest et al. 2005; Wong et al. 2008; Minagawa et al. 2011), myocardial (Takahashi et al. 2001) and cerebral ischaemia (Isozaki et al. 2011).

Other than imaging clinical application of ^62^Cu was proposed by Chan et al. (2000) Balloons filled with ^62^Cu solution have been found effective for intravascular treatment preventing coronary restenosis in porcine model.

### ^64^Cu

The most versatile isotope, ^64^Cu has found its application in: in vivo studies of copper metabolism, radiotracing biodistribution of potential therapeutics, PET imaging, cancer diagnosing and radiotherapy(preclinical and clinical trials). Although there are many methods of ^64^Cu production, the most important are those which do not require high energy beams, unattainable for typical small medical cyclotrons (Obata et al. 2003; Szajek et al. 2005; Le et al. 2009). However, such methods need enriched targets, which increase overall costs. Using natural zinc as a starting material, ^64^Cu can be produced with reasonable purity, but many highly radioactive byproducts of the reaction need to be removed and handled properly (Bonardi et al. 2003). ^64^Cu half-life allows it to be transported to locations remote of the production site, and currently this isotope is commercially available from several producers around the world.

Similarly to ^60^Cu, ^61^Cu and ^62^Cu isotopes, ^64^Cu-ATSM is subject to many ongoing research as selective tumor hypoxia imaging agent. It is in phase II clinical trials for PET/CT monitoring of therapeutic progress in patients with cervical cancer (ClinicalTrials.gov 2012). Similar compound, ^64^Cu-ATSE (Cu-diacetyl-bis(N^4^-ethylthiosemicarbazone)) (**57**), has wider tissue-oxygenation level specificity than ^64^Cu-ATSM. Increased uptake of ^64^Cu-ATSM by cell cultures occurs between oxygen concentration 0.1–0.5 %, while for the ^64^Cu-ATSE it happens between 0.1 and 5 %, which can make it more suitable imaging agent for less extreme hypoxias in myocardial and nervous tissues (McQuade et al. 2005). Because ^64^Cu is also β^−^ emitter, ^64^Cu-bis-thiosemicarbazones can be used for radiotherapy. Yoshii et al. (2011) showed that ^64^Cu-ATSM administration reduces volume and metastatic abilities of Colon-26 tumor in mice. Advantage of this treatment over other cancer therapies comes from the fact that ^64^Cu-ATSM reduced number of CD133^+^ (prominin-1 positive) cells within tumor. CD133^+^ cells contributes to ineffectiveness of cancer therapies, being chemo- and radioresistant, and also highly tumorigenic. ^64^Cu-ATSM decreases number of CD133^+^ cells not by specific interactions, but rather by accumulating within regions of tumor with high abundance of CD133^+^ cells, which results in higher doses of radiation in that areas (Yoshii et al. 2011). To increase cytotoxic effectiveness of ^64^Cu-ATSM, Aft et al. 2003 administered it together with 2-deoxy-d-glucose to mice bearing EMT-6 mammary carcinoma cell line. 2-deoxyglucose accumulates in tumor cells and potentiate effects of radiation therapy. In the study, pretreatment with 2-deoxyglucose increased tumor uptake of ^64^Cu-ATSM. Continuing daily administration (2 mg/g) of 2-deoxyglucose after single dose of ^64^Cu-ATSM increased survival time of the animals (Aft et al. 2003). Other than thiosemicarbazone ligands for ^64^Cu, based on 2-nitroimidazole (another hypoxia-selective compound), were evaluated in vivo by Engelhardt et al. (2002) and were found suitable for imaging of tumor hypoxia. More recently, Bonnitcha et al. (2010) explored an idea to conjugate thiosemicarbazones with nitroimidazoles, since these compounds have the same biological targets. Copper complexes of the ligands (**58**) synthesized by the authors showed excellent selectivity for hypoxic EMT-6 cells.
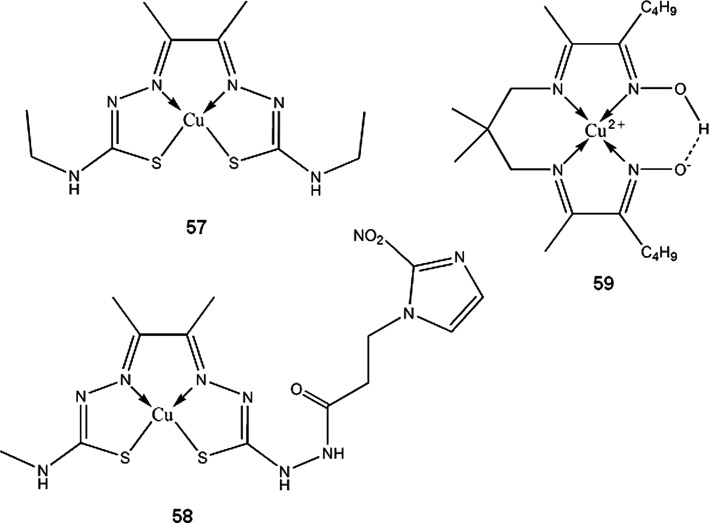


PET and SPECT (Single-Photon Emission Computed Tomography) techniques are used in mapping brain activity in behavioral studies on animals and humans. Many social behaviors cannot be monitored in immobilized test subjects. Compounds containing long living β^+^ emitters, such as ^64^Cu-PTSM, are suitable for monitoring cerebral perfusion in freely moving subjects (Holschneider and Maarek 2004). 5,13-dioximino-6,9,9,12-tetramethyl-7,11-diazaheptadeca-6,11-diene complex of copper-64 (**59**) synthesized by Packard et al. (2002) can be potentially used as myocardial perfusion imaging agent, and also for multidrug resistance screening. The complex shows tumor uptake similar to ^99m^Tc-MIBI (hexakis(2-methoxy-2-methylpropylisonitrile) technetium (^99m^Tc)), a compound used for predicting drug resistance of tumors associated with P-glycoprotein expression (Packard et al. 2002).

**Fig. 5:**
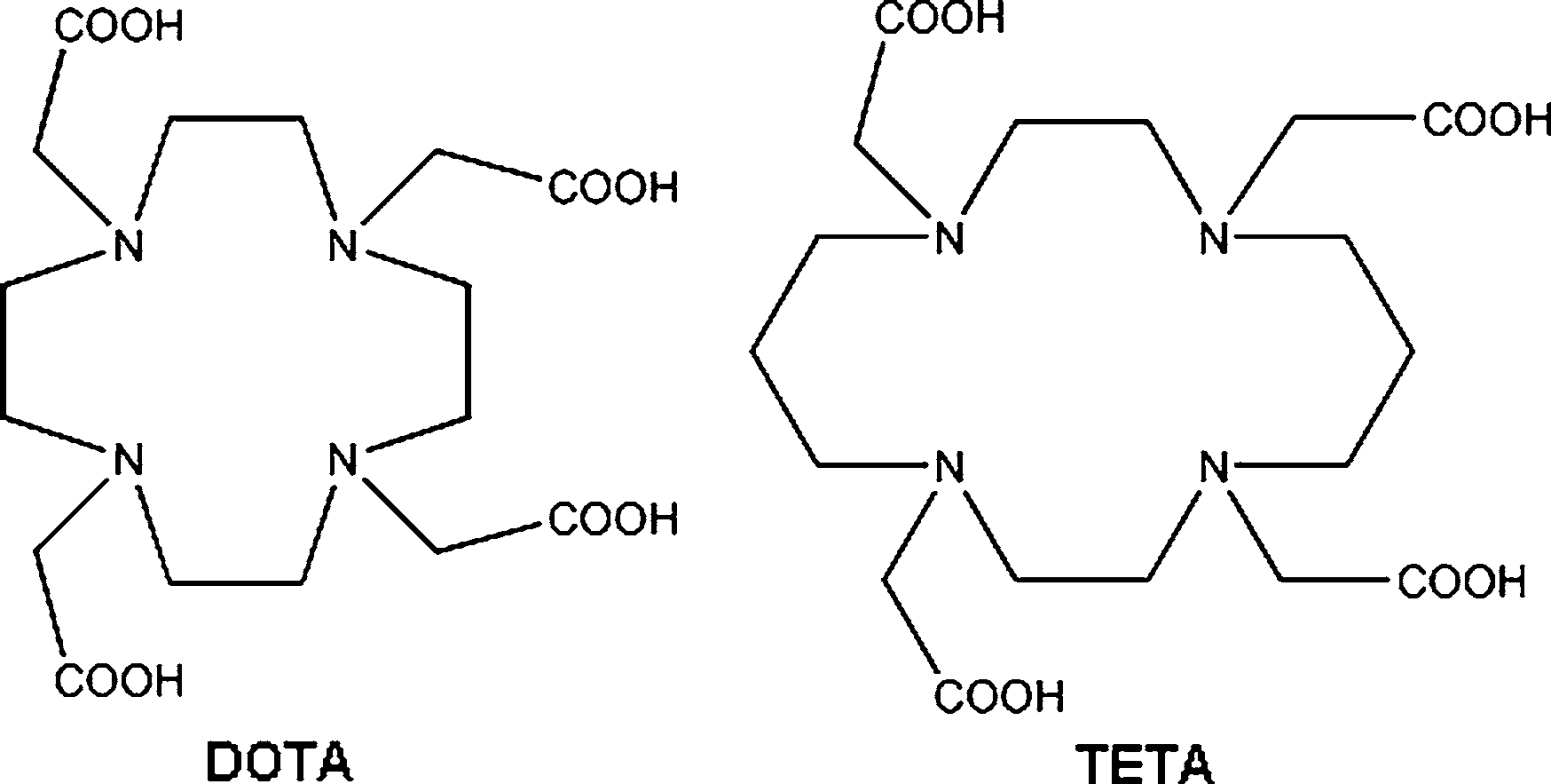
DOTA and TETA, the two most common bifunctional chelators used for labeling biomolecules

^64^Cu labeled peptides for targeted cancer therapy/imaging are one of the largest group of copper radiopharmaceuticals currently researched. They are built of a targeting peptide such as bombesin or octreotide analogue, a linker, and a bifunctional chelator (BFC), commonly tetraazamacrocycle derivate, like TETA or DOTA (Fig. 5). The peptide binds to a specific receptor expressed by cancer cells while copper isotope-BFC moiety allows localization of the tumor by positron emission detection. β^−^ radiation of ^64^Cu can also be exploited for selective irradiation of malignant cells. Attractiveness of peptides for targeted radiotherapy, in comparison to monoclonal antibodies, comes from their good tissue distribution, fast clearance, low immunogenicity, and inexpensive, automated production. By modifying amino acid composition of a peptide, one can adjust hydrophobicity, pK_a_, resistance to proteolysis, and other parameters of the peptide to form a suitable diagnostic agent. Table 2 lists the most popular peptides which were modified to be used with ^64^Cu for cancer imaging and therapy.

**Table 2:** Targeting peptides for ^64^Cu PET tracers

Peptide	Properties	Cancer type	Reference
Bombesin	Amphibian homologue of mammalian gastrin-releasing peptide (GRP)	Prostate (PC-3) Lung Breast (T-47D)	Yang et al. (2006), Hoffman and Smith (2009), Prasanphanich et al. (2009), and Lane et al. (2010)
Tyr^3^-octreotide	Somatostatin analog	Neuroendocrine tumors	Sprague et al. (2004) and Eiblmaier et al. (2007)
Arg-Gly-Asp (RGD) peptides	Ligands for α_v_β_3_ integrin, expressed during angiogenesis	Metastatic cancers	Chen et al. (2004), Wei et al. (2009), Galibert et al. (2010), and Jin et al. (2011)
VIP	Vasoactive intestinal peptide	Breast Colorectal Prostate	Thakur et al. (2004) and Zhang et al. (2007a)
PACAP	Pituitary adenylate cyclase activating peptide	Breast cancer	Zhang et al. (2007a)
α-MSH	Melanocyte stimulating hormone	Melanoma	Cheng et al. (2007) and Wei et al. (2007)
Ac-Cys-Z_EGFR:1907_	Affibody for epidermal growth factor receptor	Various types	(Miao et al. (2010)

Zhang et al. approached other than oncological use of such type of compounds. They designed a ^64^Cu-labeled peptide targeting neutrophils that can be used for non-invasive detection of acute, neutrophilic inflammation (Zhang et al. 2007c).

Monoclonal antibodies (mAbs) are vast group of biotechnologically produced proteins, with constantly rising number of applications in immunotherapy, targeted drug delivery, and in vivo*/*in vitro diagnostics. In this compounds group, we can distinguish intact immunoglobulins (murine, chimeric, humanized and human) and fragments of heavy chain antibodies (nanobodies, domain-deleted mAbs, hypervariable domain region peptides, minibodies, affibodies and other). Transforming mAbs into radiopharmaceuticals is relatively simple. When using radioisotopes such as iodine-131 or fluorine-18, small molecule labeled with atom/atoms of the isotope, coupled with a linker is attached to amino acids residues (mostly randomly) of the antibody. Similarly, if using metallic radioisotopes, a bifunctional chelator with linker is coupled to the antibody, then solution of the radioisotope salt is added to form a complex. In most cases, mAbs for radiotherapy can be formulated in a convenient form of kits, for preparation of the radiopharmaceutical right before administration to a patient (Reilly 2010). Examples of ^64^Cu-labeled antibodies for PET imaging are trastuzumab (breast cancers expressing human epidermal growth factor receptor 2 or HER2, in clinical trials) (Sampath et al. 2010; ClinicalTrials.gov 2012), 12A8 (c-kit expressing tumors) (Yoshida et al. 2011), etaracizumab (antibody against human α_v_β_3_ integrin) (Cai et al. 2006), cetuximab (targeting EGFR-epidermal growth-factor receptor expressing tumors) (Li et al. 2008).

### ^67^Cu

^67^Cu is the longest living copper radioisotope and also one of the most difficult to produce, since it requires fast neutron flux reactor or high-energy proton beams and costly ^68^Zn target (Katabuchi et al. 2008). This isotope of copper, owning to interesting decay properties, is widely acknowledged as potentially useful for radioimmunotherapy, but due to limited availability, the number of research that actually use this isotope is low, compared to other Cu isotopes. Dynamic growth of radioimmunotherapy, can increase demand for this isotope. Medvedev et al. (2012) reported an attempt to produce ^67^Cu in a larger scale, which gives perspectives for wider commercial availability of the isotope in the near future.

The possibility to change imaging agents into therapeutics is very attractive in copper radiopharmaceuticals. This can be achieved by replacing positron emitting nuclides of ^60/61/62/64^Cu with electron emitting ^67^Cu, without changing pharmacokinetics of the compounds. Since biodistribution of ^60/61/62/64^Cu-labeled substances can be monitored using PET, the data can be directly translated to ^67^Cu compounds (Cai et al. 2006). ^67^Cu is one of the best suited isotopes for radioimmunotherapy, because of its half-life long enough to allow good biodistribution within tumor (similar to biological half-life of many mAbs), relatively low gamma radiation abundance (lower whole body dose for patient and safer for medical personnel), higher tumor uptake (compared to iodine-131) and simple radiolabeling procedure (Carrel et al. 1997; Delaloye et al. 1997; DeNardo et al. 2000; Novak-Hofer and Schubiger 2002). Examples of ^67^Cu-labelled mAbs are chCE7, an anti-L1-cell adhesion molecule antibody for neuroblastoma, ovarian, and some renal carcinoma therapy (Zimmermann et al. 1999; Zimmermann et al. 2003; Knogler et al. 2007), Lym-1 for non-Hodgkin’s lymphoma (DeNardo et al. 1999; Mirick et al. 1999; DeNardo et al. 2000), C595 an anti-MUC1 mucin antibody for bladder cancer treatment (Hughes et al. 2000). Possible applications of ^67^Cu are not limited to radiotherapy; gamma radiation of the isotope can be used for Single-Photon Emission Computed Tomography (SPECT) (Engelhardt et al. 2002).

To date, no radiopharmaceutical containing copper isotope is approved for use in humans. Although many promising results were obtained during studies on Cu radiopharmaceuticals, several problems also emerged. Therefore current research have to focus on overcoming these obstacles.

- Of five discussed isotopes, only ^62^Cu can be obtained from generator. ^64^Cu and ^67^Cu have half-life long enough to be transported from remote locations, but ^60^Cu and ^61^Cu require cyclotron access. Therefore, availability of copper radioisotopes is still the main limitation for their wider application.
- High energy of emitted positrons of ^60/61/62^Cu in relative to standard PET imaging radionuclide ^18^F, is cause of the loss of spatial resolution of the resulting image. Recovery of three-dimensional data, when imaging with high-resolution PET camera, requires development of dedicated analysis algorithms (Ruangma et al. 2006; Liu et al. 2009).
- Burgman et al. (2005) found that ^64^Cu-ATSM shows cell line dependent pharmacokinetics, therefore obtained imaging data in some cases can be irrelevant to tumor hypoxia.
- Theoretical calculations made by O’Donoghue et al. (1995) indicate that tumors 2–3 mm in diameter are optimal for effective treatment with ^67^Cu. Thus, usefulness of this isotope in cancer therapy is limited only to small tumors.
- In vivo studies of first generation of Cu-radioisotope labeled peptides showed poor stability of these compounds and liberation of copper from the complexes (Mirick et al. 1999; Bass et al. 2000; Boswell et al. 2004; Sprague et al. 2006). Monocycylic tetraazamacrocycle based BFCs, such as TETA or DOTA, are not sufficiently inert in blood serum and should not be considered in designing new copper radiopharmaceuticals. Cross-bridged macrocycles are currently replacing other type chelating agents (Ma et al. 2002; Boswell et al. 2004; Anderson et al. 2008).
- Radiolabeled peptides and antibodies show high retention in kidneys which receive larger dose of radiation than other organs. To prevent kidneys damage, either dose of the radiopharmaceuticals has to be reduced, which can lead to ineffectiveness of the therapy, or additional substances reducing renal uptake need to be administered simultaneously (Vegt et al. 2010).
- Main problem of radioimmunotherapy with intact mAbs is their heterogenous biodistribution within solid tumors, resulting in insufficient dose delivered to some of the malignant cells. Therefore, it is necessary to develop other strategies for use of monoclonal antibodies in cancer radiotherapy, such as pretargeting techniques, reduction of the size of the antibody or increasing capillary permeability (Tempero et al. 2000; Goldenberg and Sharkey 2006; Reilly 2006; Thurber et al. 2008).

## Copper in nanomedicine

Past ten to twenty years are the time of rapid progress in nanotechnology and nanomedicine. Term nanotechnology generally refers to chemistry and physics of 1–100 nm sized particles, however, the term has become overused for synthesis and rational design of large molecule compounds, polymeric and colloidal materials. Reduction of size has opened new possibilities for use of metallic elements and their compounds in medicine. Metal nanosized particles or quantum dots (colloidal metal chalcogenides, consisting of core and external shell), exhibit novel physicochemical properties that cannot be observed in macroscale. Cations of metal can be complexed with multi-part macromolecular ligands, so the resulting chemical constructs can overcome limitations in distribution, bioavailability and binding specificity of simple compounds (Balogh et al. 2007; Studer et al. 2010; Gunawan et al. 2011; Webster 2011).

Biocidal properties of copper and its compounds have been known since ancient times and include antibacterial, antifungal, molluscicidal, nematocidal, antiviral and other (Borkow and Gabbay 2005). Mechanism of antimicrobial action of copper is complex and not fully understood; Cu^2+^ ions disrupt permeability of cell’s membrane, cause lipid peroxidation and proteins inactivation (Ohsumi et al. 1988; Nan et al. 2008; Raffi et al. 2010; Wu et al. 2011). Antibacterial properties of nanometer sized copper particles come mainly from ions liberation, however, the size plays important role in adsorption on bacterial cell surface (Raffi et al. 2010). It is possible to construct polymers doped with metallic or ionic copper. Such polymers can be used to make dressings, sutures, bandages and other medical materials with anti-infection, anti-inflammatory and healing-accelerating properties (Zhang et al. 2007b; Borkow et al. 2009; Grace et al. 2009; Sheikh et al. 2011). Similarly to copper nanoparticles, copper oxide nanoparticles are known to be nonspecifically cytotoxic. The activity comes from intracellular, amino acids mediated liberation of copper ions, which form complexes inducing formation of reactive oxygen species (Studer et al. 2010; Gunawan et al. 2011). Socks impregnated with copper oxide are effective in treatment of tinea pedis (fungal infection caused by *Trichophyton* genus) (Zatcoff et al. 2008). The socks can also be used for preventing so called hand and foot syndrome in capecitabine treated patients; relevant clinical studies have started (ClinicalTrials.gov 2012). Respiratory face masks with CuO offer very good protection against human influenza virus H1N1 (Borkow et al. 2010). A number of copper containing textiles and materials are already commercially available.

Most of today’s contraceptive intrauterine devices (IUDs) contain metallic copper in a form of sheet or wire. Rapid release of cupric ions in the first few days after implantation of IUD can cause adverse effects such as pelvic inflammatory disease, bleeding and expulsion (Timonen 1976; Farley et al. 1992; Mora et al. 2002). Low density polyethylene-copper nanoparticles composites show sustained, zero-order kinetic of copper ions release, therefore can be used to replace conventional IUDs (Cai et al. 2005).

Bhattacharya et al. (2006) suggested that metal nanoparticles can be used for selective precipitation and conformational alterations in proteins. They found that copper nanoparticles clusters precipitate with human hemoglobin mutant HbE, and can serve as a screening agent for hemoglobinopathies such as β-thalassemia (Bhattacharya et al. 2006). Photothermal ablation is one of the newest methods of cancer treatment. Microscopic spheres, built of dielectric core and metal shell, accumulate passively or actively (after functionalization with antibodies) in tumors, and destroy them with heat which the particles emit when excited by near infrared light. Modified gold nanoparticles are commonly researched for this purpose (Cai et al. 2008; Chen et al. 2010; Choi et al. 2011). As an alternative to costly gold, Li et al. (2010) proposed copper sulfide nanoparticles which have very good optical properties, minimal cytotoxicity and low production costs.

Quantum dots (QDs) are nanoparticles that have received much attention in medicine as tumor detection and imaging agents (Zhang et al. 2008). Coating QDs with amphiphilic polymers and functionalizing their surface with antibodies, peptides, oligonucleotides or small-molecule drugs can be done in order to facilitate targeted delivery and to reduce non-specific binding of these nanoparticles (Gao et al. 2005). To achieve quantitative imaging of tumor vasculature in deep tissues, Chen et al. (2008) successfully developed dual optical/PET tracer by functionalizing QDs with ^64^Cu-DOTA. There is little known, however, about QDs toxicity, which is an important matter, since most of QDs contain hazardous elements such as cadmium, selenium, tellurium and arsenic (Rzigalinski and Strobl 2009). Oxidation of CdSe cores and liberation of Cd^2+^ ions take place even in coated QDs (Derfus et al. 2004). Development of cadmium-free QDs could be a solution to this problem. Using copper-indium sulfide based QDs, Yong et al. (2010) achieved very promising results for novel, non-toxic, highly sensitive cancer imaging agent.

Superparamagnetic iron oxide nanoparticles are another type of nanostructures that can be functionalized in a similar manner to quantum dots. Their magnetic properties can be used for magnetic resonance imaging (MRI) of cancer. Several authors have exploited the idea of dual MRI/PET tracing to obtain complementary data on tumor localization, using ^64^Cu-DOTA labeled iron oxide particles (Jarrett et al. 2008; Lee et al. 2008).

Carbon nanotubes (CNTs) have been successfully applied in various areas of science, technology and in medicine. CNTs are very promising as multifunctional platforms for targeted therapy and imaging. A good example of such CNT construct was synthesized and tested in vivo by Liu et al. (2007). The authors used single walled CNTs coated by phospholipids-polyethyleneglycol for water solubility, functionalized with RGD peptide for targeted delivery and labeled with ^64^Cu-DOTA for PET imaging. The resulting construct showed good biodistribution and selectivity towards α_v_β_3_-positive cancer (Liu et al. 2007). CNTs do not cause acute toxicity, but there is no sufficient knowledge yet about long term exposure and distant effects on human health (Liu et al. 2008; Firme and Bandaru 2010).

Medical sensing devices are very helpful for diagnosing and for monitoring patient’s pharmacotherapy. Various authors have prepared copper nanoparticles-based electrodes for determination of glucose and other carbohydrates (Male et al. 2004; Xu et al. 2006; Jiang and Zhang 2010), drugs such as sotalol or acetaminophen (Boopathi et al. 2004; Heli et al. 2009) and amino acids (Zen et al. 2004; Dong et al. 2010). Cai et al. (2003) demonstrated that gold covered copper nanoparticles, functionalized with oligonucleotides can be used for electrochemical detection of characteristic DNA sequences present in pathogenic microorganisms or mutated genes. Nanocrystals of CuS conjoined with immunoglobulin, was a part of multiple protein detection system, developed by Liu et al. (2004) The system allows sensitive, simultaneous, electrochemical detection of proteins, and can be used to construct novel diagnosing devices.

Advances in modern polymer sciences have opened new horizons for targeted drug delivery systems and diagnostic tools development. One of the most promising and extensively studied groups of compounds are dendrimers. Dendrimers are globular shaped, branched polymers with fixed molecular weight that can be modified with various functional groups on their surface. Moreover, there are empty spaces between polymer branches that can be fitted with small molecules. There has been a lot of interest in dendrimers as potential drug carriers and artificial enzymes (Kofoed and Reymond 2005). By utilizing the ‘click chemistry’, dendrimers can be easily synthesized and functionalized. Dijkgraaf et al. (2007) used this approach to synthesize dendrimers conjoined with RGD peptides and DOTA, for use in tumor imaging after complexation with radionuclides such as ^111^In or ^64^Cu. Some dendritic copper complexes were tested for antimicrobial activity by Refat et al. (2009) and showed moderate strength of action on selected microorganisms. Poly(amidoamine)-Schiff base dendrimers synthesized by Zhao et al. (2010) form multinuclear complexes with CuCl_2_, which show good antiproliferative activity against MOLT-4 leukemia and cisplatin resistant MCF-7 breast cancer cells.

There are known several dendritic copper complexes that exhibit catalytic properties ranging from Lewis acid catalyzed addition reactions to free radical induced hydrolysis (Yang et al. 2003; Fujita et al. 2006; Kao et al. 2011). One particular example is nuclease activity of copper(II) complexes of a pyridine-modified poly(amidoamine) dendrimers. These compounds have ability to induce formation of oxygen radicals leading to cleavage of nucleic acid strand (Kao et al. 2011). Artificial nucleases can be used as anticancer drugs or for sequencing DNA and RNA. On the other hand, natural recombined nucleases, such as dornase alpha, are used in lung diseases (cystic fibrosis, chronic bronchitis), reducing viscosity of mucus in respiratory tract. An unexplored to date possibility is the use of dendritic deoxyribonucleases as potential therapeutics in aforementioned diseases.

Dendrimers can be also used as protective colloids, acting as templates, in the synthesis of copper nanoparticles with regular shape and size (Jin et al. 2008).

Another type of polymeric nanoparticles are aggregates formed by controlled self assembly of diblock amphiphilic copolymers. Many shapes can be achieved, such as spheres, rods, discs, helices, tubes, but the spheres are generally the easiest to attain and are also the most versatile. These structures can be stabilized by cross-linking and variously functionalized (O’Reilly et al. 2006). Rossin et al. (2005) synthesized shell cross-linked nanoparticles with folic acid and ^64^Cu-TETA moieties attached on the surface which can be used for early diagnosing and therapy of tumors overexpressing folate receptor.

Binding copper(II) with small peptides in some cases can induce formation of nanoaggregates of resulting complexes (Yang et al. 2008; Ren et al. 2008; Li et al. 2011). Complexing with metal ions can reinforce the biological activity of various peptides because such complexes have more rigid structure, and therefore less possible conformations (Tian and Bartlett 1996; Taraszka et al. 2000; Salvati et al. 2008). Li et al. (2011) synthesized four Cu(II)-RGD-octapeptides and found that these compounds have significantly higher anti-thrombotic activity in vivo than free RGD-octapeptides. Similar results are reported in the paper by Ren et al.(2008), in which several tripeptide-Cu(II) complexes were found to have increased thrombolytic activity both in vivo and in vitro, along with additional vasodilatation effect.

## Conclusion

Versatility of copper and its compounds has given it a strong position in development of new pharmaceuticals. Although currently there are only a few applications of Cu in medicine, numerous ongoing studies will most likely result in novel uses in the future. Copper radiopharmaceuticals will be probably the first to be approved for clinical use. Cu-containing materials and nanomaterials also hold a great promise and should soon find many applications in various fields of medicine.
